# Cerebellar disruption impairs working memory during evidence accumulation

**DOI:** 10.1038/s41467-019-11050-x

**Published:** 2019-07-16

**Authors:** Ben Deverett, Mikhail Kislin, David W. Tank, Samuel S.-H. Wang

**Affiliations:** 1 Neuroscience Institute, Princeton University, Princeton, NJ 08544 USA; 2 Rutgers Robert Wood Johnson Medical School, Piscataway, NJ 08854 USA

**Keywords:** Working memory, Decision

## Abstract

To select actions based on sensory evidence, animals must create and manipulate representations of stimulus information in memory. Here we report that during accumulation of somatosensory evidence, optogenetic manipulation of cerebellar Purkinje cells reduces the accuracy of subsequent memory-guided decisions and causes mice to downweight prior information. Behavioral deficits are consistent with the addition of noise and leak to the evidence accumulation process. We conclude that the cerebellum can influence the accurate maintenance of working memory.

## Abstract (web summary)

Disruption of cerebellar activity impairs working memory during evidence accumulation in mice. Here, the authors show that optogenetic perturbation of Purkinje cell activity disrupts the accurate accumulation of somatosensory information in working memory during perceptual decision-making.

## Introduction

The accumulation of sensory evidence in working memory is an important part of decision-making^1^. In rodents performing evidence accumulation, neuronal perturbation of specific brain regions can have distinct effects on behavior^2^. Depending on the region, perturbation can cause minimal effects^3^, it can impair functions related to decision-making^3–5^, or it can influence evidence integration in working memory^6^. Many forebrain regions implicated in evidence accumulation receive input from the lateral posterior cerebellum^7–9^, and disruption of the human cerebellum produces working memory impairments^10–13^. Given its roles in sensorimotor integration^14^ and motor preparation^15^, cerebellar output may influence the evidence accumulation process. Here we examine whether direct, temporally precise disruption of cerebellar neural activity modulates the accumulation of somatosensory evidence. We find that optogenetic manipulation of cerebellar Purkinje cells impairs decision-making by reducing the ability to effectively retain past information in working memory.

## Results

### Cue-period cerebellar disruption

**Fig. 1:**
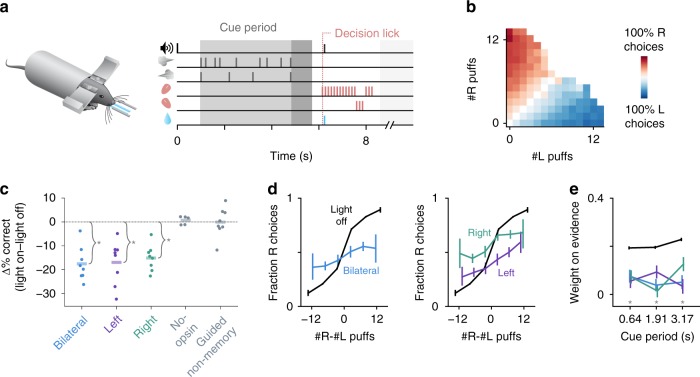
Cerebellar disruption during evidence accumulation impairs decisions. **a** Schematic of the evidence-accumulation decision-making task. In each trial, two streams of randomly timed air puffs were delivered to the left and right whiskers. After an 800-ms delay, mice licked one of two lick ports indicating the side with more cumulative puffs to receive a water reward. Gray-shaded regions from left to right: cue period, delay, intertrial interval. Decision lick: first detected lick after the delay. **b** Choice probabilities as a function of the number of left- and right-side puffs (*n* = 96,254 trials over 664 sessions in 13 mice). **c** Change in performance as a result of cue-period light delivery to the left, right, or bilateral cerebellum (*n* = 46,435 light-off trials, 5392 light-on trials, 397 sessions, 8 mice). Dots: individual mice. Lines: mean across mice. *p < 0.01 (two-tailed paired *t*-test). No-opsin: bilateral light delivery in ChR2^–^ mice (also see Supplementary Figure 3). Guided non-memory: bilateral light delivery in trials where mice were guided to lick the correct side by delivery of all-single sided puffs during the cue period and delay. **d** Psychometric curves for light-off (black) trials and light-on (colored) trials from all perturbation sessions in all experimental mice. Results are shown for bilateral (left) and unilateral (right) perturbations. Error bars: 95% CI. **e** Regression of animal choices on evidence quantity throughout the cue period for light-off (black) and light-on (colored) trials. Weights indicate the extent to which evidence was used to guide decisions, and the sum of weights is proportional to overall performance. *p < 0.01 (99% CI, light-off: 0.18–0.21, 0.18–0.21, 0.21–0.25; bilateral: 0.01–0.15, −0.03–0.11, −0.02–0.13; left: −0.02–0.13, 0.02–0.16, −0.04–0.11; right: 0–0.14, −0.05–0.08, 0.05–0.2)

We used a behavioral task for head-fixed mice in which animals accumulate sensory evidence over a period of seconds to guide decisions^16^. In each trial (Fig. 1a) the mouse is presented with simultaneous streams of randomly timed left- and right-sided whisker puffs followed by a delay, after which it licks in the direction of more puffs to retrieve a water reward. We previously showed that coarse full-session pharmacological perturbation of the lateral posterior cerebellum (crus I in rodents) alters performance in this task, and that Purkinje cell (PC) activity there encodes stimulus- and decision-related variables^16^. In the present study we trained 13 mice on this task over hundreds of behavioral sessions (Fig. 1b, Supplementary Figure 1).

To determine whether cerebellar activity can modulate the evidence accumulation process, we used time-resolved, cell-type-specific optogenetic perturbation specifically during the cue period of evidence presentation, preceding the decision. We stimulated ChR2-expressing PCs (Supplementary Figure 2), which inhibit the cerebellar output nuclei, using light delivered through optical fibers implanted bilaterally over crus I of the cerebellum. Light was delivered for the full duration of the cue period, either bilaterally or unilaterally in a randomly selected subset (15–30%) of trials over hundreds of behavioral sessions in 8 ChR2-expressing mice. Both unilateral and bilateral cerebellar perturbations led to reductions in performance (Fig. 1c–e, Supplementary Figure 4), and unilateral perturbation induced a small ipsilateral choice bias on average (Fig. 1d, Supplementary Figure 4). Using a logistic regression model to assess how animals weighted evidence to guide decisions (see Methods), we found that impaired performance was associated with downweighting of evidence throughout the cue period (Fig. 1e, Supplementary Figure 7). This cross-validated logistic regression model predicted animal choice with an accuracy of 75 ± 1% (mean ± s.d.) for light-off trials and 58 ± 2% for light-on trials. As a negative control, light delivery did not alter performance in ChR2- mice (Fig. 1c, No-opsin; Supplementary Figure 3).

In this experiment, the decision lick occurred ~1 s (1.31 ± 0.29 s, mean ± s.d.) after the end of light delivery, suggesting that the impairment did not arise from a deficit in the ability to lick. We nevertheless considered that light delivery might introduce a delayed effect that interfered with motor readout. Three measurements suggest otherwise. First, the fraction of trials in which animals made a response (in either direction) was unaffected by the perturbation (98.6 ± 1.8% mean ± s.d. in light-on trials vs 99.7 ± 0.3% in light-off trials; *p* = 0.11, two-tailed paired *t*-test). Second, the latency from the end of the delay period to the decision lick was indistinguishable between light-on and light-off trials (578 ± 222 ms mean ± s.d. light-off vs 595 ± 332 ms light-on; *p* = 0.19 bilateral, *p* = 0.84 left, *p* = 0.14 right, two-tailed paired t-test within subjects; Supplementary Figure 5). Finally, light delivery did not influence the ability to make directed decision licks in trials where mice were cued which direction to lick with all-unilateral puffs during the cue period and delay (Fig. 1c, Guided non-memory; Supplementary Figure 3). Therefore, cerebellar disruption during the cue period affected not the ability to lick but rather one or more aspects of the preceding process.

### Sub-cue-period cerebellar disruption

**Fig. 2:**
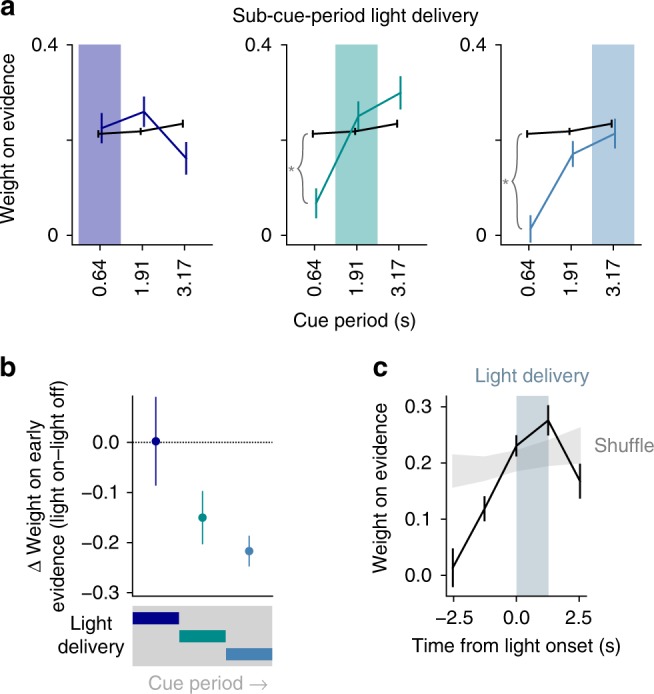
Cerebellar disruption influences weighting of past evidence. **a** Regression of animal choices on evidence quantity for light-off (black) and light-on (colored) trials (*n* = 32,311 light-off trials, 5669 light-on trials, 285 sessions, 8 mice). Weights indicate the extent to which evidence was used to guide decisions, and the sum of weights is proportional to overall performance. Colored shading indicates the time of light delivery. Error bars: s.e.m. of regression weights. *p < 0.01 (99% CI on first bin, light-off: 0.19–0.23; light-on middle third: −0.01–0.15; light-on last third: −0.06–0.09). **b** Change in weight on evidence in the first third of cue period as a function of when light was delivered during the cue period. Data points and error bars show mean ± s.e.m. across mice. **c** Evidence weight as a function of time relative to the onset of light delivery, with all cue-period light delivery conditions included (see Methods). Shuffle: light delivery time labels were shuffled before regression. Error bars: bootstrap s.d.

The observed impairment could be explained by a variety of mechanisms, including alteration of the weight of incoming stimuli (e.g. sensory gating or attentional disruption), impairment of the retention of past stimulus information, or interference with translation of accumulated information into directed motor actions^15^ (Supplementary Figure 6). We tested these alternatives by introducing additional trials in which light was delivered during a subsection of the cue period (Fig. 2). By regressing animal choice on evidence strength throughout the cue period (as in Fig. 1e), we quantified which specific cues animals remembered and incorporated into their choices, lending insight into the contents of their working memory when light was applied. Importantly, this approach differentiates scenarios that appear similar with simpler analyses, such as one in which light resets the animal's retention of accumulated evidence vs. one in which accumulation is intact but light prevents the animal from executing the desired lick (Supplementary Figure 6).

Surprisingly, mice had no difficulty using the evidence presented concurrent with light delivery, but they did have difficulty retaining evidence that had been previously presented (Fig. 2a–c, Supplementary Figure 7). In the most extreme case, light delivery in the final third caused mice to completely discount evidence from the first third of the cue period (Fig. 2a right panel, first weight 95% CI: −0.04 to 0.07). In other words, light delivery in the middle and final third did not cause uniform effects across all trials, but instead selectively altered behavior in those trials where evidence was strong near the start of the cue period, prior to light delivery. In additional separate trials with light delivery during the post-evidence delay period, mice downweighted evidence throughout the entire preceding cue period (Supplementary Figure 8).

### Drift-diffusion behavioral modeling

These results suggest that cerebellar perturbation influenced behavior by altering how mice integrate and retain evidence information over time. We further tested this hypothesis by fitting our data to an established drift-diffusion framework that explicitly models the incremental integration of pulses of evidence to form decisions^17^. Crucially, this model differentiates impairments in evidence integration and storage per se (e.g., leakiness of evidence from memory) from non-specific impairments such as decision lapses that occur when animals fail to translate accumulated information into the proper action (Supplementary Movie 1, Supplementary Figure 9). The model achieves specificity by taking advantage of the broad statistical distribution of stimulus timings available from thousands of trials.

**Fig. 3:**
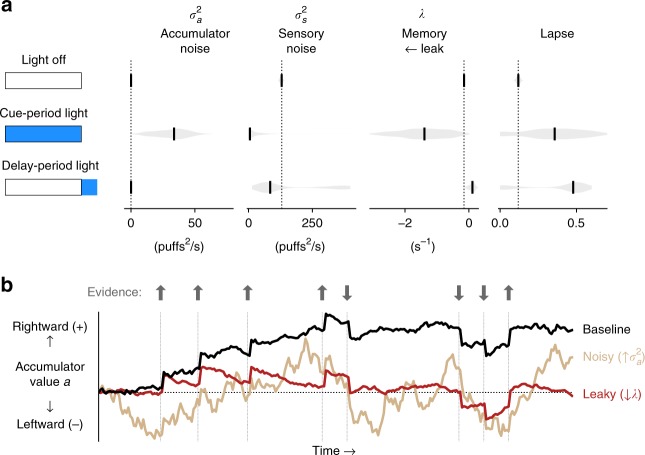
Fits to a drift-diffusion model reveal specific deficits in evidence accumulation. **a** Best-fit drift-diffusion model parameters in different light delivery conditions (schematics on left indicate light delivery condition, with the box denoting the cue period and blue shading denoting light delivery). Fits were computed multiple times for each condition using random subsets of the data to assess the reliability of the best-fit parameters (see Methods). Black vertical ticks indicate the median best-fit parameter across fit repetitions. Gray shading represents the distribution of fit parameters across repetitions. Vertical dotted lines denote best-fit values in the light-off condition. **b** Visualization of the drift-diffusion model. The model's accumulator value *a* is shown as it evolves over time in a single behavioral trial. Colored lines demonstrate how the trajectory of *a* is qualitatively altered by changes in specific parameters. Arrows and associated vertical lines indicate pulses of evidence. See also Supplementary Movie 1

Our model estimated parameters quantifying accumulator noise $(\sigma _a^2)$, sensory noise $(\sigma _s^2)$, memory leak or instability (*λ*), left-right bias, and a lapse rate. We fit all trials pooled across mice for the baseline light-off condition (*n* = 56,550 trials), full-cue-period light delivery (*n* = 6,394 trials), and delay-period light delivery (*n* = 2,369 trials), and we assessed the goodness of fit using cross-validated metrics (see Methods, Supplementary Table 1). Fits to light-off trials (Fig. 3a, top row, Supplementary Table 1) demonstrate that at baseline mice performed evidence accumulation using strategies similar to mice, rats, and humans performing similar visual and auditory evidence accumulation tasks^17,18^. Specifically, mice exhibited small values for accumulator diffusion noise and lapse rate, and leaky accumulation (*λ* < 0) consistent with the regression analysis (Supplementary Figure 1b).

When light was delivered for the full cue period (Fig. 3a, second row, Supplementary Table 1), behavior was characterized by an increase in $\sigma _a^2$ the diffusion noise in the accumulation process, and a decrease in λ, indicative of leakiness in evidence integration. Strikingly, the decay time constant $\left( {\tau = \frac{1}{\lambda }} \right)$ of accumulated evidence in working memory decreased approximately tenfold, from 6.7 s in the baseline condition to 0.72 s with light delivery. Therefore, cerebellar disruption impaired the noise and persistent time course of accumulated working memory contents (Fig. 3b, Supplementary Movie 1). In contrast, when cerebellar activity was perturbed during the delay (Fig. 3a, bottom row, Supplementary Table 1), behavior was likely best explained by an increase in lapse rate, which may also have been present with full-cue-period perturbation, though these lapse rate alterations were not statistically significant. Increases in lapse rate are consistent with disruptions to accumulated information or to translation of that information into actions.

### Whisker measurements

Given that the cerebellum is involved in sensorimotor circuits related to whisking, we asked whether our perturbation influenced whisker movement during the task. Using behavioral movies acquired during the experimental sessions, we measured and analyzed whisker movement in the different perturbation conditions (Supplementary Figure 10, Supplementary Movie 2). In all conditions we observed a ~200-ms increase in whisker movement following the bilateral puffs that were delivered at the start and end of the cue period. Consistent with previous studies^14,19^, we observed a similar transient increase in whisker movement following the onset of light delivery, and a smaller one that sometimes followed the offset of light delivery. These whisker movements may be related to attentional modulation at salient events in the trial and also may be the result of modulating whisker-related sensorimotor circuits^14,19^.

## Discussion

Our principal finding is that cerebellar perturbation influences sensory evidence accumulation by impairing the ability to maintain evidence in working memory. These results are consistent with clinical memory impairments observed after cerebellar lesions^10,11^, cerebellar roles in sensorimotor integration^14,20^ and models of cerebellar function in working memory^12,13,21–23^.

In our previous study using the same behavioral task^16^, pharmacological disruption with muscimol produced a deficit similar to the one observed here, causing compression of the psychometric curve, i.e. decreasing the ability of evidence to influence the animal's choice. Here, our temporally resolved perturbations and modeling reveal the detailed behavioral properties of this deficit: cerebellar disruption impairs the dynamics of the accumulation process, introducing noise and leak into working memory storage. However, it does not reduce sensory input gain, because regression weights and sensory noise parameters were normal during light delivery. In other words, cerebellar perturbation does not disrupt the ability to sense or encode new sensory information; rather, it disrupts the ability to retain prior information. Additionally, delay-period perturbation and full-cue-period perturbation may increase the lapse rate (i.e. probability of a random decision), consistent with a failure to maintain decision-related^3,24^ and possibly motor-preparatory information^15,25^ in memory following evidence accumulation.

These specific effects on accurate and stable memory maintenance differ from the effects seen in perturbations of other brain regions^3–6^. In a similar mouse evidence accumulation task in virtual reality^18^, an optogenetic survey of 29 dorsal neocortical regions revealed widespread regional involvements in successful task performance, but no region that was necessary for retaining prior evidence^5^. In a rat auditory evidence accumulation task, disruption of the posterior parietal cortex had no substantial effects on behavior^3^, while disruption of a frontal cortical region affected a post-categorization function distinct from the accumulation of information in memory^3,4,24^. Of all regions studied to date, only disruption of the anterior dorsal striatum appears to influence the evidence integration process itself^6^. Therefore, the cerebellum may make contributions to evidence accumulation that are distinct and complementary to the functions of previously studied brain structures.

It is likely that cerebellar activity exerts its influence through communication with forebrain regions^8,26^. The lateral posterior cerebellum makes anatomical and functional connections bidirectionally with almost every region previously implicated in evidence accumulation and perceptual decision-making^1,2,5–9,26–29^. Furthermore, cerebellar disruption in rodents can modulate long-timescale forebrain neural activity^14,15,25,27^. Prominent theories of decision-making postulate that decisions are made by the evolution of persistent neocortical activity over seconds-long periods^1,2^. Our results are consistent with the idea that cerebellar inputs play a necessary role in supporting this persistent activity^15^.

Contributions to decision-making may vary by cerebellar region. All three cerebellar nuclei express preparatory neural activity for movement^15,25^. Our region of focus, the ansiform area of the lateral posterior cerebellum (crus I in rodents)^30^, projects primarily to the dentate nucleus^28^. However, in a recent study of motor preparation for licking, disruption of the fastigial but not the dentate nucleus affected behavioral choices^15^. Our task is more cognitively demanding, requiring long-timescale manipulation of working memory contents. This task demand may recruit activity in the dentate nucleus, which receives input from the location of our perturbation. Therefore, recent reports of cerebellar neural activity in premotor^15^ and working memory^16^ contexts may reflect multiple cerebellar roles that support distinct cognitive and sensorimotor functions depending on the demands of the task.

Our findings provide evidence for a hypothesized role of the cerebellum in working memory^22,23^. These results may help account for the many clinical findings linking cerebellar activity to working memory and decision-making in humans^8,11,31,32^. Future work should address the extent to which our findings generalize to other sensory and decision modalities, as well as the detailed mechanisms by which the cerebellum contributes to brainwide dynamics underlying decision-making, evidence accumulation, and working memory.

## Methods

### Mice

Experimental procedures were approved by the Princeton University Institutional Animal Care and Use Committee (protocol 1943-16) and performed in accordance with the animal welfare guidelines of the National Institutes of Health. Data for the behavioral task came from 13 mice (5 female, 8 male, 8–25 weeks of age during experiments) of genotypes *Pcp2-Cre* for Purkinje-cell specificity and *Ai27D* for channelrhodopsin-2 (8 animals *Pcp2-Cre* x *Ai27D*, 5 animals *Ai27D*) acquired from The Jackson Laboratory, Stock #010536 (RRID:IMSR_JAX:010536) and #012567 (RRID:IMSR_JAX:012567), respectively. Experimenters were blinded to the genotypes of the mice for the duration of the experiments. Data for electrophysiology experiments came from an additional 3 mice of genotype *Pcp2-Cre* x *Ai27D*. Mice were housed in a 12-h:12-h reverse light:dark cycle facility, and experiments were performed during the dark cycle. During the experimental day, mice were housed in darkness in an enrichment box containing bedding, houses, wheels (Bio-Serv Fast-Trac K3250/K3251), climbing chains, and play tubes. At other times, mice were housed in cages in the animal facility in groups of 2–4 mice per cage. Mice received 1.0–1.5 mL of water per day. Body weight and condition was monitored daily.

### Surgical procedures

Mice were anesthetized with isoflurane (5% for induction, 1.0–2.5% for maintenance) and underwent surgical procedures lasting 2–4 h. Two ~500 μ${\mathrm{m}}$ diameter craniotomies were drilled over the cerebellum, one over each hemisphere, directly posterior to the lamboid suture and ~3.6 mm lateral to the midline in either direction. Ferrule implants were constructed as in Spart et al.^33^ with 400-μm-diameter optical fiber (Thorlabs FT400EMT) glued to 1.25-mm OD stainless steel ferrules (Precision Fiber Products MM-FER2007-304-4500) using epoxy (Precision Fiber Products PFP 353ND). Ferrules were positioned over each craniotomy with the fiber tip at the surface of the dura mater, and Vetbond (3 M) was applied surrounding the exposed fiber. Dental cement (C&B Metabond, Parkell Inc.), darkened by mixing with India ink (Koh-I-Noor #3080-4), was then applied to secure the ferrule to the skull. In some mice, separate implants were placed over neocortex for other experiments. When animals were not engaged in experiments, optical implants were protected using ceramic ferrule sleeves (Precision Fiber Products SM-CS, 1.25-mm ID, 6.6-mm length). Implants were cleaned before each behavior session using a fiber optic cleaning kit (Thorlabs CKF). A custom-machined titanium headplate^34^ was cemented to the skull using dental cement (C&B Metabond, Parkell Inc.). All animals were given buprenorphine (0.1 mg/kg body weight) and rimadyl (5 mg/kg body weight) after surgery and were given at least 5 days of recovery in their home cages before the start of experiments.

### Behavior

Mice were trained to perform an evidence-accumulation decision-making task^16^. Briefly, head-fixed mice were seated in tube for 1-h behavioral sessions consisting of 200–300 trials. In each trial, independent streams of randomly timed 40-ms air puffs (2.5 Hz, minimum 200 ms interpuff interval) were delivered to the left and right sides over the course of a 3.8-second or 1.5-second cue period (duration chosen randomly with 0.85 and 0.15 probability, respectively). After a delay of 800 ms (or in ~10% of early sessions, 200 ms), lick ports were advanced into the reach of the animal, and animals licked to the side with the greater number of puffs to retrieve a water reward. The animals decision was interpreted as the side licked first, regardless of subsequent licks. Guided non-memory trials had the same structure except puffs were delivered only on a single side throughout the cue period, and regular 2.5 Hz guide puffs were delivered during the delay; choice was again defined as the side of the first lick (and in guided trials a reward was delivered in all cases independent of choice). The behavioral apparatuses were controlled by custom-written Python software (https://github.com/wanglabprinceton/accumulating_puffs).

### Optogenetics

Light for optogenetic stimulation was produced by two 470-nm LEDs (Thorlabs M470F3, one for each implant) each powered by an LED driver (Thorlabs LEDD1B). Fiber optic patch cables (Thorlabs M98L01) carried light from the LEDs to the ferrule implants, where they were connected via custom-machined black Delrin sleeves. Light was delivered through 400-μm diameter optical fibers in 5-ms pulses at 50 Hz (generated by Master-8, A.M.P.I.) with an intensity of 3–15 mW/mm^2^. Based on published results^35–38^ we estimate that the light emitted from each fiber illuminated a roughly spherical region of tissue <1 mm in diameter, corresponding to a large fraction of cerebellar crus I. Light delivery was triggered via electrical signals sent by the behavioral control software through a DAQ card (National Instruments, NI PCI-MIO-16E-4). Cue period light was delivered over the entire cue period through the left, right, or both implants. Sub-cue-period light was delivered bilaterally to both implants for one third of the cue period, and delay period light was delivered bilaterally to both implants for the entire 800-ms delay period or for the first 200 or 500 ms. Light delivery trials were interleaved with light-off trials and were selected randomly with a uniform probability (ranging from 15–30%) throughout the session. All analyses compare light-off and light-on trials only from behavioral sessions in which light was delivered.

### Electrophysiology

Single-unit recordings in 3 awake Pcp2-Cre-Ai27D mice were performed using borosilicate glass electrodes (1B100F-4, World Precision Instruments) with 1- to 2-μm tips and 3 to 12 MΩ impedance, fabricated on a pipette puller (P-2000, Sutter Instruments Co.) and filled with sterile saline. Electrical signals were amplified with a CV-7B headstage and Multiclamp 700B amplifier, digitized at 10 kHz with a Digidata 1440A and acquired in pClamp (Axon Instruments, Molecular Devices) in parallel with TTL pulses from a signal generator (Master-8), which was used to synchronize recording and optical stimulation. Light was delivered through a ferrule implant identical to those used in behavior experiments, positioned above an open craniotomy and connected to a fiber-coupled LED (M470F3, Thorlabs) with a TTL-controlled driver (LEDD1B, Thorlabs). The fiber optic was always moved independently of the recording electrode using a second motorized micromanipulator (MP-225; Sutter Instrument Co.). The optical stimulation parameters were the same as those used in the behavioral experiments. Spike detection was performed using custom code written in MATLAB 2017b.

### Histology

Animals were deeply anesthetized and then transcardially perfused using a peristaltic pump with phosphate buffered saline (PBS) followed by chilled 10% formalin (Fisher Scientific). Brains were extracted from the skull after perfusion, postfixed overnight at 4 °C, cryoprotected in 30% sucrose in PBS, embedded in O.C.T. compound 4585 (Tissue-Plus, Fisher HealthCare) and stored at −80 °C until sectioning. 50-μm thick sagittal sections were cut with a Leica CM3050 S cryostat. To remove the cryoprotective solution, sections were washed with PBS. Sections were mounted on slides and covered with Fluoroshield anti-fade reagent with DAPI (Sigma). Images were acquired on an inverted fluorescent microscope (Nikon Eclipse Ti) using NIS-Elements AR software. Image processing was performed in Python.

### Software

Data analyses and figure creation were performed using custom code written for Python 3.6 (code available at https://github.com/bensondaled/puffsopto), which makes use of Numpy 1.14.3^39^, Scipy 1.0.0^40^, Pandas 0.23.4^41^, Matplotlib 2.2.2^42^, IPython 6.1.0^43^, Scikit-learn 0.19.1^44^, and Statsmodels 0.9.0^45^.

### Performance and psychometrics

Data for performance and psychometric measures were obtained only from trials in the final stages of the task and not from the preceding stages during the shaping procedure. Performance, psychometric, and regression analyses contain only trials in which mice made decision licks such that incorrect trials correspond to licks in the wrong direction and never the absence of a decision lick. Optogenetic analyses compare light-off and light-on trials only from sessions in which light-on trials were delivered and only from trials with the primary 3.8-second cue period. Confidence intervals on fractions of correct or left/right-choice trials were computed by the Jeffreys method for binomial confidence intervals. The meta-mouse psychometric curve in Supplementary Figure 1a consists of pooled trials from all mice and was fit to a four-parameter logistic function of the form

1 $$y(x) = y_0 + \frac{A}{{1 + e^{ - \frac{{(x - x_0)}}{b}}}} .$$

### Behavior regression analysis

To determine the dependence of animal choice on stimuli in different temporal bins of the cue period, we performed a regression-based analysis. Data for regression analysis consisted of trials with a cue period duration of 3.8 s. Logistic regressions were performed with animal decision on a trial-by-trial basis as the predicted variable. The input for each trial was a vector of values corresponding to the difference in right vs left puffs in temporally uniform bins of the cue period; i.e. bin edges of 0-1.27 s, 1.27-2.53 s, and 2.53-3.8 s. The model was of the form

2 $$ln\frac{p}{{1 - p}} = \beta _1E_1 + \beta _2E_2 + \beta _3E_3$$

where *β*_*i*_ is the weight on the i-th bin, and *E*_*i*_ is the #R-#L puffs quantity in the i-th bin. The regression was fit and confidence intervals obtained using Statsmodels 0.9.046. The choice prediction accuracy of the model was evaluated using k-fold cross-validation with scikit-learn 0.19.1^44^ with k = 3. The light-delivery-aligned regression in Fig. 2c was computed by performing the regression analysis on each perturbation condition separately, then averaging weights across conditions aligned to light onset, wherever these weights existed. For example, the weight following light offset is the mean regression weight at that time point from the first- and middle-third light delivery conditions. Error bars were computed using a bootstrap approach: for each regression fit, a random sample of trials was selected with replacement from the set of trials to be fit, and the analysis was run on these trials. This procedure was repeated 100 times and error bars were computed as the standard deviation of the resulting weights across runs.

### Simulations for regression analyses

For all simulations in Supplementary Figure 7, we used the full baseline dataset of 48,239 non-manipulation trials delivered to animals during real experiments. In light-off and no-impairment simulations (left column and top row in Supplementary Figure 4), simulated decisions were sampled trial-by-trial from the empirical psychometric curve exhibited by the trained animals. For light delivery conditions (remainder of panels), the decisions were also simulated in this way, but with the addition of simulated perturbation-like interventions, as follows: (1) in the sensation/attention impairment scenario, for each trial, stimuli coinciding with light delivery were given half the magnitude of all other stimuli, then the cumulative evidence was summed for the trial yielding a new effective total #R-#L value, from which a decision was drawn using the empirical psychometric curve like above. (2) in the retention impairment scenario, for each trial, stimuli preceding light delivery were given half the magnitude of all other stimuli and the same procedure was applied. (3) in the action impairment scenario, for each trial stimuli were summed (i.e. accumulated) normally and decisions were drawn as in the no-impairment condition, but then the decision was stochastically switched to the opposite side with a probability inversely proportional to the time until the decision lick, emulating a failure to execute the decision that matches the agent's internal accumulated memory. Regressions were performed on each resulting simulation dataset in the same manner as the data figures.

### Whisker movement measurement

To measure whisker movement, we used behavioral movies acquired below the animal's face in all behavioral sessions (Supplementary Movie 2). For each behavioral session, regions of interest were manually selected corresponding to the locations of the left and right whiskers. Then, for each pair of sequential frames throughout the movie, the pixel-wise optical flow was computed using a standard freely available optical flow estimation package^46^ (code at https://github.com/pathak22/pyflow). The mean absolute optical flow values within the left and right whisker regions of interest were summed to produce the measure of whisker movement for each time point. An example of the output is shown in Supplementary Movie 2.

### Drift-diffusion modeling

Our model is based on the one presented by Brunton et al.^17^. In each trial, an accumulator value *a*(*t*) tracks the level of evidence presented in the trial so far, with right-sided stimuli corresponding to positive deflections and left-sided stimuli to negative deflections. When the trial ends, the choice is defined as the sign of *a*, positive for rightward choices and negative for leftward choices. $\sigma _a^2$ is a diffusion constant that parameterizes noise in *a*. $\sigma _s^2$ parameterizes noise associated with single left or right puffs. *λ* parameterizes drift in the memory *a*. When *λ* < 0, the accumulator *a* drifts towards 0, causing earlier evidence to influence the decision less than later evidence, a property that is often called leakiness. When *λ* > 0, the accumulator *a* drifts further from 0, causing earlier puffs to influence the decision more than later puffs, often called instability. These features are implemented by the model

3 $$da = \sigma _adW + \lambda adt + \left( {\delta _{t,t_R} \cdot \eta _R - \delta _{t,t_L} \cdot \eta _L} \right)$$

where $\delta _{t,t_{R/L}}$ are delta functions at the puff events, η_*R*/*L*_ are i.i.d. Gaussian variables drawn from *N*(1, *σ*_*s*_), and *dW* is a white-noise Wiener process. At time *t* = 0, the value of *a* is set to 0. In addition, a bias parameterizes an offset in *a* and a lapse rate parameterizes the fraction of trials on which a random response is made (the probability of a rightward decision at the end of a trial where *a* > 0 is 1–0.5*lapse). Ideal performance is characterized by an accumulator value *a* = #*R*-#*L* puffs, which would be achieved by setting the following parameter values: *λ* = 0, $\sigma _a^2 = 0$, $\sigma _s^2 = 0$, bias = 0, lapse = 0.

The model was fitted using automatic differentiation as in Yartsev et al.^6^ (code found at https://github.com/misun6312/PBupsModel.jl). This approach computes the approximate probability distribution of the accumulator value a on a trial-by-trial and time point-by-time point basis, yielding a measure of the model likelihood at the end of each trial, i.e. the probability of making a left/right choice given a particular parameter set. Automatic differentiation was then used to find the parameters that maximize the model likelihood over all fit trials. Fits were included in analyses only if the resulting Hessian matrix of the model likelihood with respect to the model parameters was positive semidefinite. Each model was fit 1000 times, initializing with random values for each parameter and omitting a random 20% of trials in each repetition. The median parameter values and confidence intervals were assessed across fit repetitions.

The model choice prediction accuracy was evaluated using a cross-validation procedure: following every fit on 80% of the data, we used the best-fit parameters *θ* and the model-likelihood function to compute a predicted choice in each of the 20% held-out trials (selecting a right choice if *p*(*R*|*θ*) > 0.5 and a left choice otherwise). In addition, for the fits to light-off, full-cue-period light, and delay-period light data, we considered models with the lapse or bias parameter omitted and computed the Bayesian Information Criterion^47^ of the model fit:

4 $${\mathrm{BIC}} = {\mathrm{ln}}\,L - \frac{{k\,{\mathrm{ln}}\,n}}{2}$$

where *k* is the number of parameters fit, *n* is the number of trials used to fit the model, and L is the likelihood of the data under the model given the best-fit parameters. In Supplementary Table 1 we show the BIC for each of these fits relative to the full model, where 0 corresponds to the BIC of the full model for a given condition and positive values indicate a favored model relative to the full model.

For drift-diffusion model simulations, the demonstration in the second row of Supplementary Figure 9 was produced as follows: random subsamples (*n* = 500 subsamples, 10,000 trials each) were collected from the behavioral dataset without perturbation (i.e. light-off). A simulated perturbation was then introduced by choosing a random 25% of trials and replacing the true animal choice with the opposite of the true choice. This reflects the concept of a lapse: i.e. an impairment in selecting the desired response, and specifically one that is not tied to the timing or quantity of accumulated evidence information. Each of the 500 subsamples of trials with the perturbation applied was then fitted to the drift-diffusion model using the same methods as the data fitting in Fig. 3.

The trials shown in Supplementary Movie 1 and Fig. 3b were generated as follows: a single trial with 5 left puffs and 3 right puffs was produced, and the accumulator value *a* throughout the trial was calculated by running the model (equation in the Drift-Diffusion Modeling section above) in discrete time steps of 15 ms. For the baseline case, parameters were chosen to be similar to the empirically fit light-off behavioral data (Supplementary Table 1). The leaky, noisy, and lapse conditions were simulated by altering those parameters and rerunning the simulation. Playback was slowed for visualization purposes.

### Reporting Summary

Further information on research design is available in the Nature Research Reporting Summary linked to this article.

## Supplementary information

Supplementary Information

Peer Review

Reporting Summary

Description of Additional Supplementary Files

Supplementary Movie 1

Supplementary Movie 2

## Data Availability

The datasets generated and analyzed during the current study are available from the corresponding author upon reasonable request. Figure data can be found at https://github.com/bensondaled/puffsopto.

## Code availability

Code used for data acquisition is available at https://github.com/wanglabprinceton/accumulating_puffs. Code used for analysis is available at https://github.com/bensondaled/puffsopto.

## References

[1] Gold JI, Shadlen MN (2007). The neural basis of decision making. Annu. Rev. Neurosci..

[2] Brody C. D. & Hanks T. D. Neural underpinnings of the evidence accumulator. *Curr. Opin. Neurobiol.* **37**, 149–157 (2016). 10.1016/j.conb.2016.01.003 PMC5777584 26878969

[3] Erlich J. C., Brunton B. W., Duan C. A., Hanks T. D. & Brody C. D. Distinct effects of prefrontal and parietal cortex inactivations on an accumulation of evidence task in the rat. *eLife*, **4**, e05457 (2015). 10.7554/eLife.05457 PMC4392479 25869470

[4] Hanks TD (2015). Distinct relationships of parietal and prefrontal cortices to evidence accumulation. Nature.

[5] Pinto L., Tank D., Brody C. & Thiberge S. Widespread cortical involvement in evidence-based navigation. In *Cosyne Abstracts* (2018).

[6] M. M. Yartsev, T. D. Hanks, A. M. Yoon, and C. D. Brody. Causal contribution and dynamical encoding in the striatum during evidence accumulation. *eLife*, **7**, e34929 (2018). 10.7554/eLife.34929 PMC6147735 30141773

[7] Prevosto V, Graf W, Ugolini G (2010). Cerebellar inputs to intraparietal cortex areas LIP and MIP: functional frameworks for adaptive control of eye movements, reaching, and arm/eye/head movement coordination. Cereb. Cortex.

[8] Strick PL, Dum RP, Fiez JA (2009). Cerebellum and nonmotor function. Annu. Rev. Neurosci..

[9] Bostan AC, Strick PL (2018). The basal ganglia and the cerebellum: nodes in an integrated network. Nat. Rev. Neurosci..

[10] Schmahmann JD, Sherman JC (1998). The cerebellar cognitive affective syndrome. Brain: J. Neurol..

[11] Kansal K (2017). Structural cerebellar correlates of cognitive and motor dysfunctions in cerebellar degeneration. Brain.

[12] Ravizza SM (2006). Cerebellar damage produces selective deficits in verbal working memory. Brain.

[13] Ferrari C (2018). TMS over the cerebellum interferes with short-term memory of visual sequences. Sci. Rep..

[14] Proville RD (2014). Cerebellum involvement in cortical sensorimotor circuits for the control of voluntary movements. Nat. Neurosci..

[15] Gao Z (2018). A cortico-cerebellar loop for motor planning. Nature.

[16] Deverett B, Koay SA, Oostland M, Wang SSH (2018). Cerebellar involvement in an evidence-accumulation decision-making task. eLife.

[17] Brunton BW, Botvinick MM, Brody CD (2013). Rats and humans can optimally accumulate evidence for decision-making. Science.

[18] Pinto L. et al. An accumulation-of-evidence task using visual pulses for mice navigating in virtual reality. *Front. Behav. Neurosci.* **12**, 36 (2018). 10.3389/fnbeh.2018.00036 PMC5845651 29559900

[19] Brown S. T. & Raman I. M. Sensorimotor integration and amplification of reflexive whisking by well-timed spiking in the cerebellar corticonuclear circuit. *Neuron* **99**, 564–575 (2018). 10.1016/j.neuron.2018.06.028 PMC6367942 30017394

[20] Ishikawa T., Shimuta M. & Hausser M. Multimodal sensory integration in single cerebellar granule cells in vivo. *eLife*, **4**, e12916 (2015). 10.7554/eLife.12916 PMC4798944 26714108

[21] Sheu Y-S, Liang Y, Desmond JE (2019). Disruption of cerebellar prediction in verbal working memory. Front. Hum. Neurosci..

[22] Popa L. S., Hewitt A. L. & Ebner T. J. The cerebellum for jocks and nerds alike. *Front. Syst. Neurosci.*, **8** (2014). 10.3389/fnsys.2014.00113 PMC4060457 24987338

[23] Ito M (2008). Control of mental activities by internal models in the cerebellum. Nat. Rev. Neurosci..

[24] Piet AT, Erlich JC, Kopec CD, Brody CD (2017). Rat prefrontal cortex inactivations during decision making are explained by bistable attractor dynamics. Neural Comput..

[25] Chabrol F p., Blot A. & Mrsic-Flogel T. D. Cerebellar contribution to preparatory activity in motor neocortex. *bioRxiv*, 335703 (2018). 10.1016/j.neuron.2019.05.022 PMC6693889 31201123

[26] Buckner RL, Krienen FM, Castellanos A, Diaz JC, Yeo BTT (2011). The organization of the human cerebellum estimated by intrinsic functional connectivity. J. Neurophysiol..

[27] Parker KL (2017). Delta-frequency stimulation of cerebellar projections can compensate for schizophrenia-related medial frontal dysfunction. Mol. Psychiatry.

[28] Sun Lynn W (2013). Viral and non-viral tracing of cerebellar corticonuclear and vestibulorubral projections in the mouse. Open J. Neurosci..

[29] Koay S. A., Thiberge S. Y., Brody C. D. & Tank D. W. Neural correlates of cognition in primary visual versus neighboring posterior cortices during visual evidence-accumulation-based navigation. *bioRxiv*, (2019).

[30] Luo Y. et al. Lobular homology in cerebellar hemispheres of humans, non-human primates and rodents: a structural, axonal tracing and molecular expression analysis. *Brain Struct. Funct.*, 1–24 (2017). 10.1007/s00429-017-1436-9 28508291

[31] Blackwood N (2004). The cerebellum and decision making under uncertainty. Cogn. Brain Res..

[32] Reeber S. L., Otis T. S. & Sillitoe R. V. New roles for the cerebellum in health and disease. *Front. Syst. Neurosci.*, **7** (2013). 10.3389/fnsys.2013.00083 PMC3827539 24294192

[33] Sparta DR (2011). Construction of implantable optical fibers for long-term optogenetic manipulation of neural circuits. Nat. Protoc..

[34] Dombeck DA, Khabbaz AN, Collman F, Adelman TL, Tank DW (2007). Imaging large-scale neural activity with cellular resolution in awake, mobile mice. Neuron.

[35] Barbara TD (2013). Cerebellar Purkinje cell activity drives motor learning. Nat. Neurosci..

[36] Tsubota T, Ohashi Y, Tamura K, Sato A, Miyashita Y (2011). Optogenetic manipulation of cerebellar purkinje cell activity in vivo. PLoS ONE.

[37] Yizhar O, Fenno LE, Davidson TJ, Mogri M, Deisseroth K (2011). Optogenetics in neural systems. Neuron.

[38] Kruse W (2014). Optogenetic modulation and multi-electrode analysis of cerebellar networks in vivo. PLoS ONE.

[39] Oliphant T (2006). A Guide to NumPy..

[40] Jones E., Oliphant T., Peterson P. & others. *SciPy: Open Source Scientific Tools for Python* (2001).

[41] McKinney W. in *Proceedings of the 9th Python in Science Conference* (Stefan van der Walt, and Jarrod Millman, eds) 51–56 (2010).

[42] Hunter JD (2007). Matplotlib: A 2d graphics environment. Comput. Sci. Eng..

[43] Perez F, Granger BE (2007). IPython: a system for interactive scientific computing. Comput. Sci. Eng..

[44] Pedregosa F (2011). Scikit-learn: machine learning in python. J. Mach. Learn. Res..

[45] Seabold S. & Perktold J. Statsmodels: Econometric and statistical modeling with python. In *9th Python in Science Conference* (2010).

[46] Pathak D., Girshick R., Dollar P., Darrell T. & Hariharan B. Learning Features by Watching Objects Move. In *CVPR* (2017).

[47] Schwarz G (1978). Estimating the dimension of a model. Ann. Stat..

