# Supplementary material for: Cerebellar disruption impairs working memory during evidence accumulation
Source: Nat Commun. 2019 Jul 16;10:3128. doi: 10.1038/s41467-019-11050-x (PMC6635393; doi:10.1038/s41467-019-11050-x)
Supplement: Supplementary file 1 — Supplementary Information [file 41467_2019_11050_MOESM1_ESM.pdf]

Supplementary materials for

Cerebellar disruption impairs working memory during evidence accumulation

by Deverett et al. 2019

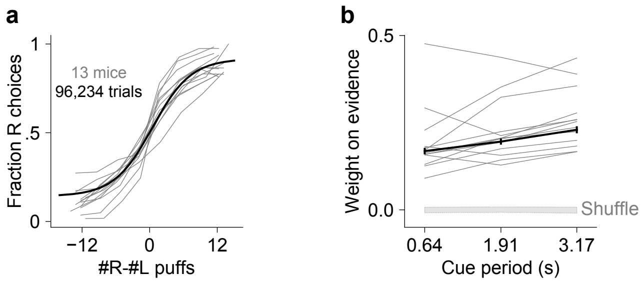

Supplementary Figure 1: Performance in the somatosensory evidence accumulation task. (a) Psychometric curves for all individual mice (gray lines) and psychometric fit to the meta-mouse (black) consisting of all trials from all mice (n=96,254 trials over 664 sessions in 13 mice). Error bars: 95% CI. (b) Regression analysis demonstrating how mice weight evidence throughout the cue period to guide decisions. Weights indicate the extent to which evidence was used to guide decisions, and the sum of weights is proportional to overall performance. Upward slope indicates a slight tendency to weight later evidence more heavily than earlier evidence (error bars show 95% CI), which would be predicted by leaky integration of stimuli. Gray lines: individual mice. Black line: meta-mouse. Bottom gray shading: 95% CI when choice was shuffled across trials.

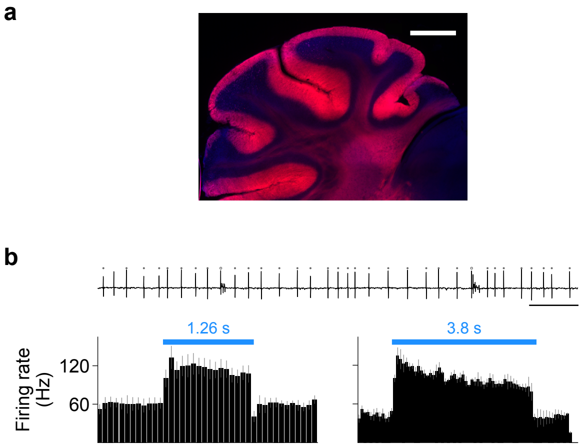

Supplementary Figure 2: Optogenetic manipulation of cerebellar Purkinje cells. (a) ChR2 expression in cerebellar Purkinje cells. Scale bar: 0.5 mm. Blue: DAPI, red: tdTomato fused to ChR2. (b) Top: extracellular electrical recording from an example Purkinje cell. Circles: simple spikes (filled) and complex spikes (open). Scale bar: 50 ms. Bottom: simple spike firing rate in response to light delivery (blue bars) of different durations. Bars show mean ± s.e.m. of firing rate (n=10 cells, 3 mice). Bin width: 80 ms.

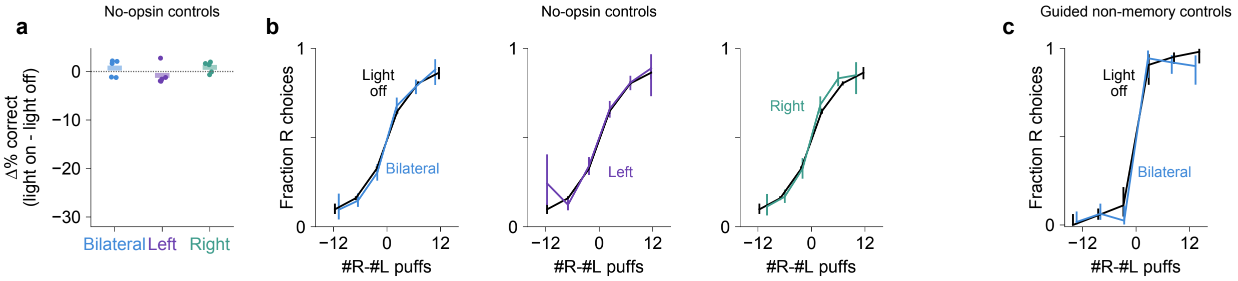

Supplementary Figure 3: Light delivery has no behavioral effect in ChR2- mice or in non-memory control trials. (a) Change in performance with cue-period light delivery, as in Figure 1c, for no-opsin control mice (n=15,281 light-off trials, 3,883 light-on trials, 118 sessions, 5 mice). Dots: individual mice. Horizontal lines: mean across mice. Data are not significantly different from zero; left to right: p=0.42, 0.42, 0.17 (two-tailed paired t-tests). Error bars: 95% CI. (b) Psychometric curves as in Figure 1d for control mice. Error bars: 95% CI. (c) Psychometric curves for experimental mice in trials requiring no memory, where mice were guided to lick the correct side by delivery of all single-sided puffs during the cue period and delay (n=558 light-off trials, 397 bilateral light-on trials, 8 mice). Error bars: 95% CI.

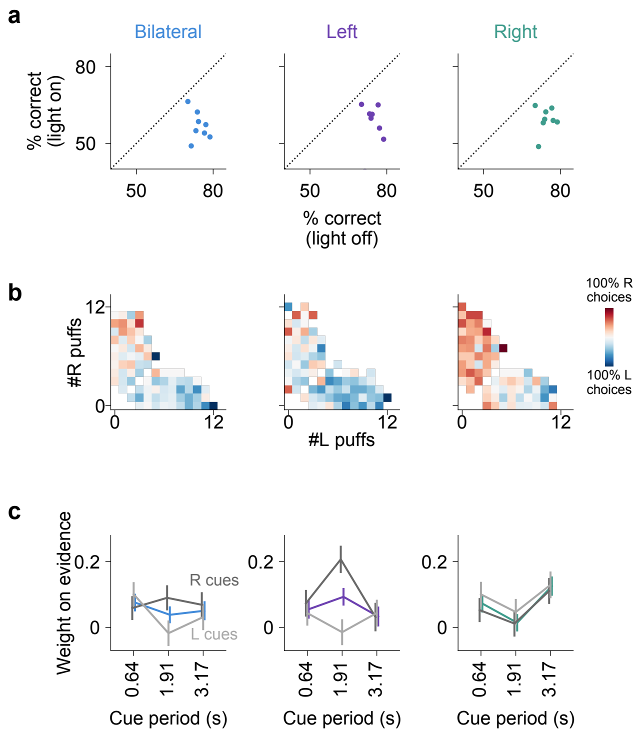

Supplementary Figure 4: Behavioral measures in the full-cue-period perturbation. (a) Relationship between performance (percent correct trials) in the full-cue-period light-off vs light-on conditions. Each dot corresponds to one subject. (b) Choice probabilities as a function of the number of left- and right-side puffs, as in Figure 1b, for the light-on conditions. (c) Regression analyses as in Figure 1e (colored lines), and the same analysis performed using only left-sided (light gray) or right-sided (dark gray) puff counts as regressors.

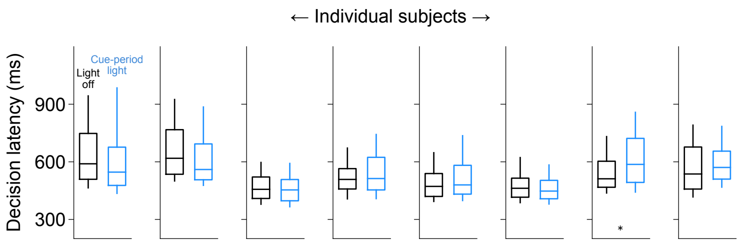

Supplementary Figure 5: Decision latencies in individual subjects. Each panel corresponds to an individual subject and displays the distribution of decision latencies (i.e. time from end of delay to decision lick) in the light-off and full-cue-period light-on conditions. Box: lower and upper quartile; whiskers: 10-90th percentile; horizontal line: median. *: $p<0.05$, two-tailed t-test comparing light-on and light-off data.

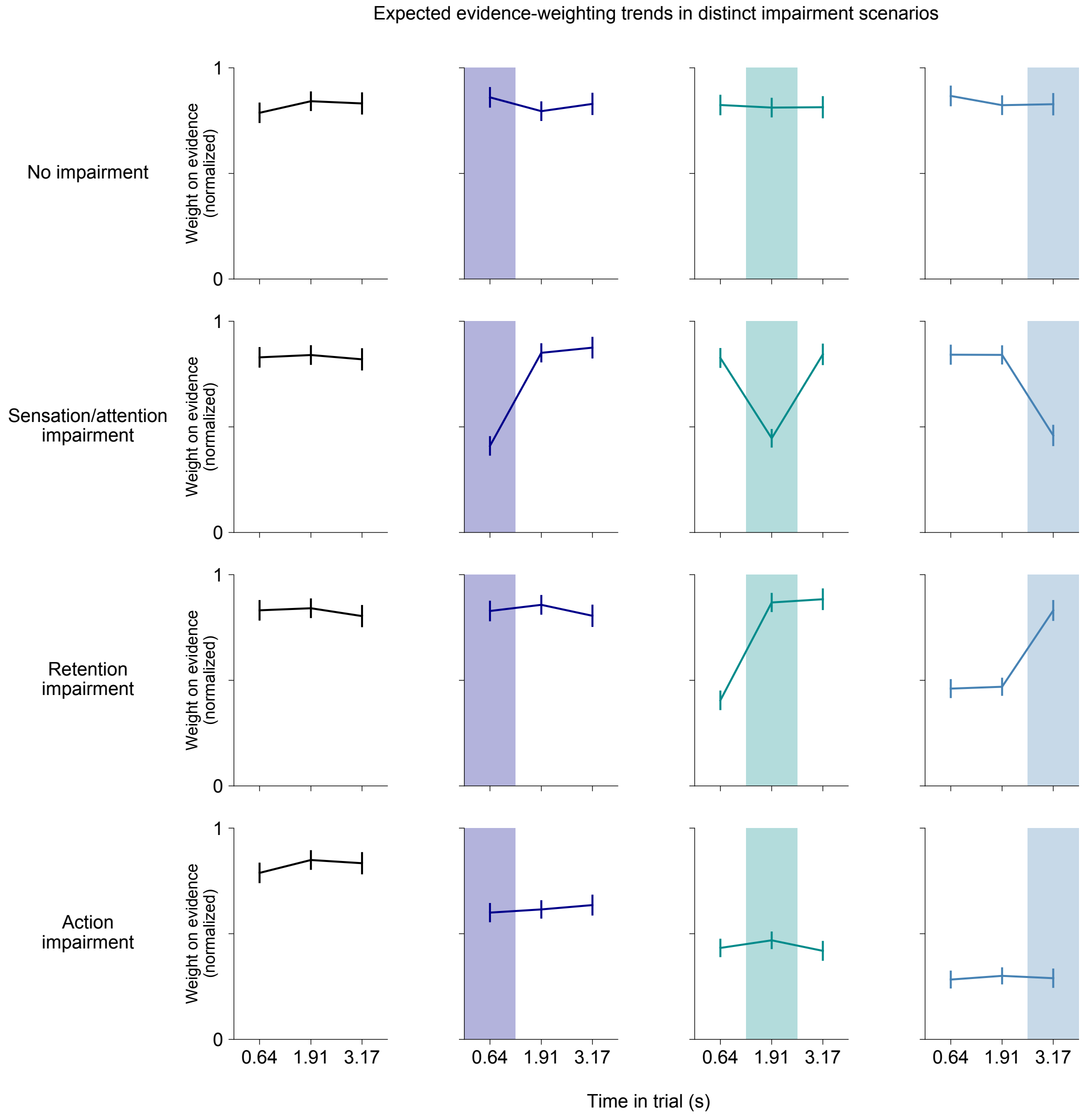

Supplementary Figure 6: Demonstration of how regression analysis can differentiate distinct perturbation effects. Simulations of different perturbations were performed. Trials and associated animal decisions were drawn from the baseline light-off behavioral dataset. Then animal decisions were perturbed according to particular rules for four example scenarios, presented in the four rows respectively. Each row shows four light delivery conditions from left to right, indicated by shading as in Figure 2 (leftmost column: light off). First row: light delivery causes no impairment. Second row: light delivery impairs animals' ability to sense, encode, or attend to stimuli delivered concurrently with the light. Third row: light delivery impairs the retention of previously accumulated information (i.e. memory). Fourth row: evidence accumulation is intact but light delivery causes a failure to translate the accumulated information into an action, with increasing probability as the decision approaches. All regressions were performed and presented as in Figure 2a. Regression weights indicate the extent to which evidence was used to guide decisions, and overall performance in any given scenario is proportional to the sum of weights. Error bars: 95% CI.

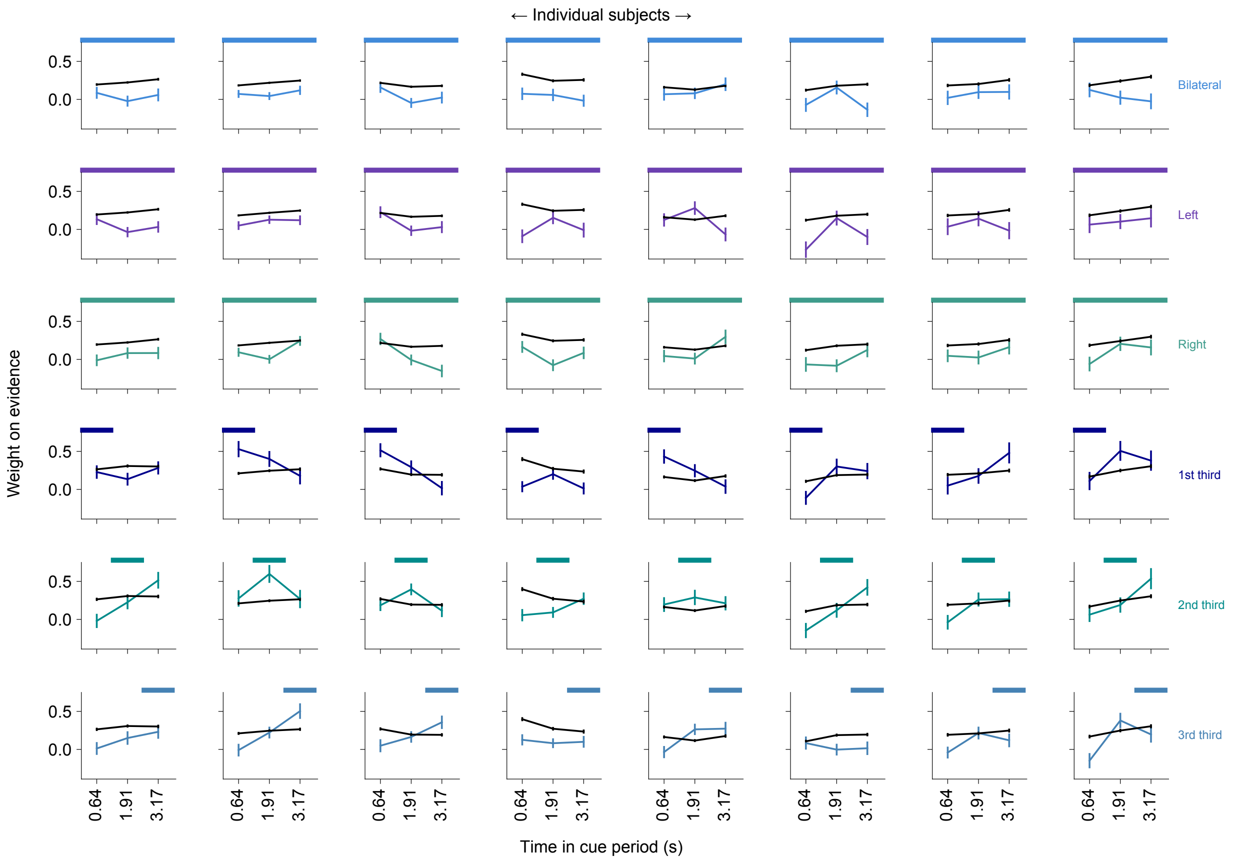

Supplementary Figure 7: Regression analysis on individual subjects. Each column of panels corresponds to an individual subject, and each row of panels corresponds to a light-delivery condition. Colored lines above panels (and labels in rightmost column) indicate the time of light delivery. Black lines: light-off condition. Colors and error bars use the same conventions as in Figure 2.

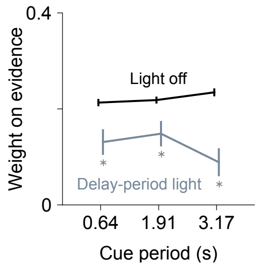

Supplementary Figure 8: Delay period perturbation. Regression analysis as in Figure 1e for all light-off (black) and light-on (gray) trials (n=28,959 light-off trials, 2,060 light-on trials, 256 sessions, 8 mice). Weights indicate the extent to which evidence was used to guide decisions, and the sum of weights is proportional to overall performance. *: $p<0.05$ (95% CI, light-off: 0.2-0.23, 0.2-0.23, 0.22-0.25; light-on: 0.08-0.18, 0.1-0.2, 0.03-0.15).

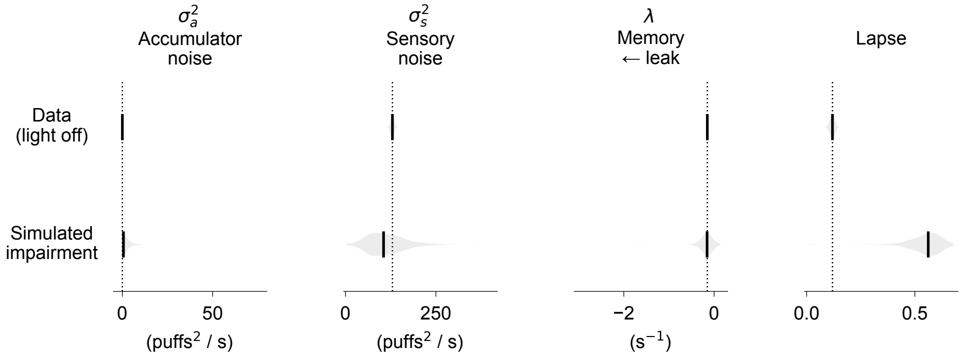

Supplementary Figure 9: Simulated lapses demonstrate the specificity of model parameters. Subsamples of trials were randomly sampled from the baseline no-perturbation behavioral dataset, and a specific decision impairment was simulated: in a scenario where stimulus accumulation in working memory is intact but animals stochastically fail to translate this information into a directed action, one would observe a random subset of trials in which decisions are opposite of the accumulated information in memory. We therefore simulated the impairment by inverting the animals choice on a random subset of trials in these sampled datasets. These trials were then fit to the model in the same manner as the real data (see Methods for additional details). As expected, the model captured the impairment as an increase in lapse rate with no effect on other parameters. This exemplifies the power of the model to identify specific deficits, confirming that the alterations in other parameters with cerebellar perturbation (Figure 3) are not explained by lapses in animals ability to report the information accumulated in working memory. Display conventions are the same as those in Figure 3.

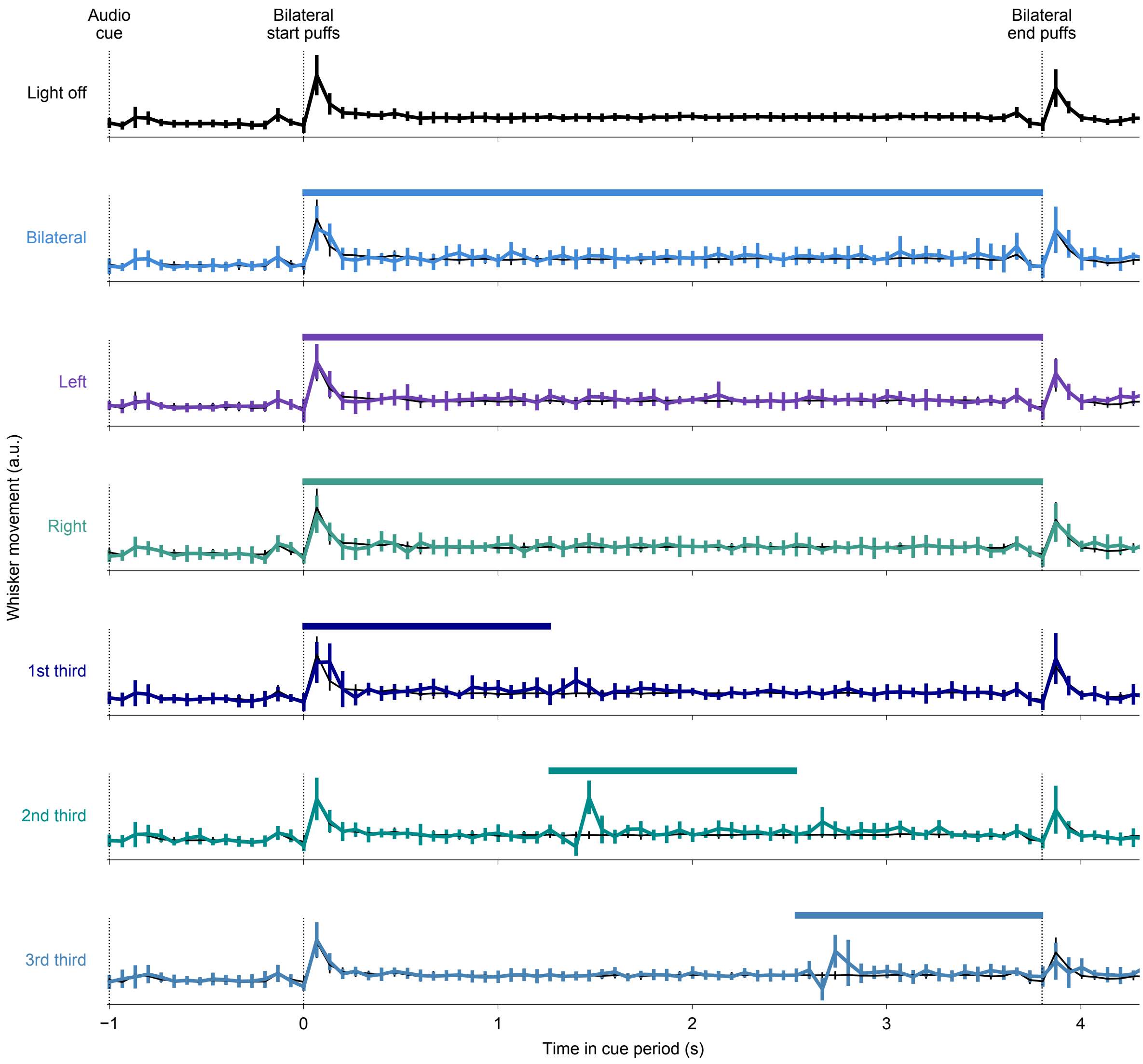

Supplementary Figure 10: Measurement of whisker movements. Whisker movement was measured in behavioral movies using a region-of-interest optical flow analysis (see Supplementary Movie 2 and Methods). Each row corresponds to a single light-delivery condition and displays the mean ± s.d. whisker movement across all subjects. Colored bars along the tops of panels (and labels at left) indicate light delivery. The black line corresponds to the light-off condition and is shown in all panels for reference. Dashed vertical lines: points of interest during the trial; the cue period spans the range between the starting and ending bilateral puffs.

Supplementary Table 1: Best-fit drift diffusion model parameters (95% CI)

	σ_a^2 (puffs^2 / s)	σ_s^2 (puffs^2 / s)	λ (s^{-1})	bias	lapse	Cross-validated choice prediction accuracy (mean ± sd)	Relative BIC
Light off	0.01 (0.00 – 0.08)	129.84 (120.62 – 139.08)	-0.15 (-0.17 – -0.13)	-0.43 (-0.46 – -0.39)	0.12 (0.10 – 0.14)	72.7 ± 0.4%	0
Full cue period light	33.67 (6.72 – 53.63)	5.54 (0.03 – 150.07)	-1.39 (-2.77 – -0.24)	0.48 (0.23 – 1.10)	0.36 (0.00 – 0.66)	58.2 ± 1.2%	0
Delay period light	0.00 (0.00 – 1.48)	84.75 (37.30 – 397.57)	0.11 (-0.09 – 0.24)	1.93 (1.37 – 2.55)	0.48 (0.00 – 0.56)	65.4 ± 2.0%	0
(fit w/out lapse) Light off	0.00 (0.00 – 0.00)	172.87 (169.30 – 176.33)	-0.19 (-0.22 – -0.17)	-0.40 (-0.43 – -0.37)	n/a	72.7 ± 0.4%	1
(fit w/out lapse) Full cue period light	49.58 (39.16 – 64.61)	6.29 (0.04 – 65.93)	-2.70 (-3.15 – -2.37)	0.26 (0.17 – 0.39)	n/a	58.2 ± 1.2%	4
(fit w/out lapse) Delay period light	0.12 (0.00 – 2.69)	376.95 (331.20 – 399.98)	-0.03 (-0.19 – 0.09)	1.71 (1.21 – 2.26)	n/a	65.4 ± 1.9%	3
(fit w/out bias) Light off	0.02 (0.00 – 0.32)	129.22 (119.37 – 137.75)	-0.14 (-0.16 – -0.12)	n/a	0.13 (0.10 – 0.14)	72.7 ± 0.4%	-64
(fit w/out bias) Full cue period light	31.53 (5.16 – 46.85)	15.84 (0.05 – 153.55)	-1.23 (-2.49 – -0.21)	n/a	0.39 (0.03 – 0.72)	57.9 ± 1.1%	-1
(fit w/out bias) Delay period light	0.10 (0.00 – 1.88)	160.55 (46.92 – 399.53)	0.11 (-0.08 – 0.25)	n/a	0.33 (0.00 – 0.54)	65.1 ± 1.7%	-4
First third cue period light	1.06 (0.00 – 3.45)	55.43 (25.61 – 94.81)	0.10 (0.03 – 0.18)	-0.43 (-0.72 – -0.16)	0.24 (0.16 – 0.30)	74.1 ± 1.9%	n/a
Middle third cue period light	0.03 (0.00 – 0.73)	110.83 (88.49 – 138.23)	-0.46 (-0.54 – -0.37)	-0.07 (-0.17 – 0.03)	0.05 (0.00 – 0.14)	71.6 ± 1.9%	n/a
Final third cue period light	5.45 (0.96 – 10.49)	44.32 (2.73 – 99.34)	-0.53 (-0.69 – -0.34)	0.45 (0.29 – 0.77)	0.33 (0.18 – 0.43)	64.8 ± 2.0%	n/a
