# Supplementary material for: Cerebellar disruption impairs working memory during evidence accumulation
Source: Nat Commun. 2019 Jul 16;10:3128. doi: 10.1038/s41467-019-11050-x (PMC6635393; doi:10.1038/s41467-019-11050-x)
Supplement: Supplementary file 4 — Description of Additional Supplementary Files [file 41467_2019_11050_MOESM4_ESM.pdf]

Description of Additional Supplementary Files

File Name: Supplementary Movie 1
Description: **Visual demonstration of the drift diffusion model parameters affected by cerebellar perturbation.** Four consecutive scenarios are shown, all displaying the same behavioral trial (5 left puffs, 3 right puffs; the correct choice is left). All four scenarios display the model's accumulator value (i.e. working memory trace of stimulus information) over time as the stimuli are presented. The moving circle and white line display the evolving value of the accumulator. The color of the circle at any given moment indicates the sign of the accumulator and thus the side the agent would select if the decision occurred at that moment (in the absence of lapses). The arrows indicate puff (evidence) events, and the choice is indicated at the end of the trial by the flashing box. In each scenario, the meaning of one specific parameter is demonstrated.

File Name: Supplementary Movie 2
Description: **Measurement of whisker movement.** Whisker movement was extracted from behavioral movies using an optical-flow region-of-interest analysis (see Methods). The side plots show the whisker movement trace from the left and right sides, and the bottom plot shows the summed left- and right-sided whisker movement.
