# Supplementary material for: Cerebellar disruption impairs working memory during evidence accumulation
Source: Nat Commun. 2019 Jul 16;10:3128. doi: 10.1038/s41467-019-11050-x (PMC6635393; doi:10.1038/s41467-019-11050-x)
Supplement: Supplementary file 2 — Peer Review [file 41467_2019_11050_MOESM2_ESM.pdf]

Reviewers' Comments:

Reviewer #1:
Remarks to the Author:
In this manuscript, Deverett et al. use optogenetic perturbations in a left-right discrimination task to study the role of the cerebellum in evidence accumulation. They demonstrate that activation of Purkinje cells in the hemispheres on either side during the cue sampling period disrupts performance in the task. Activation during a sub-interval of the cue epoch selectively disrupts the effect of cues that occur prior to this interval. Fitting a drift-diffusion model to the data suggests that cue-period inactivation increases the noise in the decision signal and increases the leak, while delay-period inactivation disrupts the effect of this decision signal on the behavioral choice.

In general, the experiments are well-designed and the figures presented clearly. The results represent an advance over the authors' previous work, which used pharmacology and calcium imaging in Purkinje cells (ref. 17), but did not reveal the trial- or trial-epoch-specific effects of cerebellar perturbation.

Major points

How frequently did animals fail to lick in either direction? The authors should indicate, for each condition, the fraction of trials with no licks. Is it the case that the "Fraction R choices" plots (e.g. fig. 1d) display data only from trials in which licks occurred?

One might expect the perturbations to change the reaction time statistics, but no difference in mean reaction time between control and laser was observed the the pooled data. Did any individual animals exhibit reaction time differences? The standard deviation is rather large; does this reflect differences between animals? Furthermore, the standard deviation appears to be higher for laser (332 ms) than control trials (222 ms). It would be useful to see the mean and standard deviation of the reaction time for control and laser trials for each individual animal.

How well did the logistic regression model predict behavior? The authors should assess this on a subset of data not used to fit the model. More details should be provided for the logistic regression analysis, and the model should be written explicitly in the methods. Were three independent variables (number of peri-cue bins) used, and if so, what were the bin edges? The caption for fig. 1e states that success rate is proportional to the sum of the logistic regression weights; why is this true? It would be useful to see the weights for all animals plotted individually for fig. 2 and 1e. Finally, please label the x axis at the three plotted points, rather than 0 and 0.25 s.

Some aspects of the drift-diffusion model should be explained and justified more thoroughly:

(1) The goodness-of-fit of the drift-diffusion model should be characterized in some way, and should be assessed on a test set not used to fit the model.

(2) Is left/right bias the difference between ηR and ηL, or is there an additional bias parameter? If the latter is the case, it should be included in the equation on line 360.

(3) What is the probability of a rightward lick if $a(t) > 0$ at the end of the trial? It appears to be 1-.5*lapse, but this should be stated.

(4) Why is a lapse rate parameter needed? It seems that larger values of this parameter reflect a

weaker influence of a(t) on behavior. So why not fit the model without a lapse rate and see if the goodness-of-fit is poorer in the perturbation conditions?

(5) If the model was fit using the BPupsModel package, the github page should be linked. The fitting procedure should be explained in detail.

Minor points

In fig. 1b (or the supplement), please show a similar panel for laser-on trials.

In fig. 1c (or the supplement), the authors should also show laser-on vs laser-off as a scatter plot, in addition to showing the difference.

From the discussion: "cerebellar disruption impaired the noise and stability." Would a different word than "stability" be better, as $\lambda < 0$ will increase (not decrease) the stability of a(t) around 0?

Reviewer #2:
Remarks to the Author:
This ms follows up on a recent study by the same lab that describes a role for the lateral cerebellum in an evidence accumulation task in mice. Whereas the past study inactivated lateral cerebellum w muscimol, the present study takes advantage of temporal control afforded by optogenetics to attempt to gain insights into the specific role of the cerebellum in this task. The issue of cerebellar roles in non-motor function is timely and of general interest. Because similar decision-making tasks have been well studied in the context of numerous forebrain structures in rodents, the present study could (potentially) elucidate interactions between the cerebellum and forebrain.

1. An important methodological concern is whether the effects of the manipulation are truly related to evidence accumulation or other decision-related processes versus a host of other possibilities more in line with traditional roles of cerebellum in sensori-motor functions. The authors argue that motor behavior is unaffected by the stimulation but they should also check whether the optogenetic stimulation has effects on the movement and position of the whiskers during the stimulation, as might be expected based on previous studies (for example from the Lena group). Video monitoring of whiskers was performed previously for muscimol inactivation but should be reported again here for the optogenetic stimulation.

Another recent study (Brown and Raman) show that air-puffs evoke reflexive whisker protraction in awake mice. Moreover, opto activation of Purkinje cells alters such movements (as well as firing of PCs). If both the stimulus used and the optpo manipulation are engaging/altering whisker sensori-motor loops, the interpretation of the results could be very different (i.e. the mice are not just passively integrating sensory evidence).

2. A recent paper (Gao et al. Nature) report a necessary role for the medial cerebellar nucleus in a whisker-based working memory task. The paper shows that the lateral nucleus is not needed. How does this square with the present results? Presumably, the cortical area activated here projects to the lateral nucleus. A weakness of the present study is that no attempts were made to map the cerebellar regions involved in the task. As far I can tell, the previous paper from the same group did not do this either. Especially in light of the Gao paper, what, if anything, can we conclude regarding how different regions of the cerebellum might contribute to the author's task? The interpretation would be different

if activating wide swaths of the cerebellum had exactly the same effect.

3. I have some questions about the main conclusions. The previous paper ended with: "For example, output signals from the cerebellum may be combined with signals in sensory circuits to control the input gain of sensory information into accumulators elsewhere in the brain. This model would be consistent with observations of cerebellar involvement in gating sensory information (Apps et al., 1997; Ozden et al., 2012) and inputs to working memory (Baier et al., 2014; Sobczak-Edmans et al., 2016). In a second possibility, the cerebellum may modulate dynamics of the accumulation process. Finally, cerebellar signals may modulate activity that converts the accumulator value into a decision. Such a post-categorization influence has been observed in prefrontal regions during evidence accumulation (Erlich et al., 2015). Detailed inactivation studies with high spatial and temporal precision can resolve these alternatives."

I feel that the present ms should give me the answer but I am not sure what it is. This could be a problem with the writing, the experiments, the reviewer or some combination. Perhaps the authors can clarify?

More specifically, I am not sure why in the model-based analysis of figure 3a the experiments in which light was only given during part of the cue period are not included? The authors seem to interpret the analysis in fig. 3 to indicate impairment in integration but earlier in the paper they say that surprisingly, integration is fine when the light is on. How are these consistent? Also, Figure 3 shows a change in the lapse rate in both experimental conditions, however, the authors seem to ignore this, concluding that the deficits in the two conditions are caused by separate processes.

4. I found the discussion section of the paper too brief and too vague. Given the large amount of past work in this area, I see this as a major deficiency. Readers will want to understand how the cerebellum fits into existing schemes regarding the neural basis for decision making. Presumably, a major motivation of the study is to understand the interrelations between the cerebellum and forebrain regions, motivating the use of a task that has been heavily studied in the context of the neocortex, as well as the striatum.
Vague statements such as "The behavioral effects we characterized here were not observed with perturbations of other brain regions in similar paradigms 3–5,7 ." should be expanded upon such that the reader can understand in reasonable detail how the present results compare to ostensibly very similar manipulations performed in very similar tasks in other brain regions, including PPC, FOF, and dorsal striatum. Even if the answers are not clear, stating clearly what the remaining questions are will be helpful.

The authors should also comment on how the result of the optogenetic activations used here compare to results they obtained previously using muscimol. This is an important issue given many recent results showing that different manipulations can give unexpectedly different results in the same task.

Response to Reviews

We thank the reviewers for their thoughtful and constructive critiques.
Below we address each point, with **reviewer text in bold** and author responses in blue.

Reviewer #1 (Remarks to the Author):

In this manuscript, Deverett et al. use optogenetic perturbations in a left-right discrimination task to study the role of the cerebellum in evidence accumulation. They demonstrate that activation of Purkinje cells in the hemispheres on either side during the cue sampling period disrupts performance in the task. Activation during a sub-interval of the cue epoch selectively disrupts the effect of cues that occur prior to this interval. Fitting a drift-diffusion model to the data suggests that cue-period inactivation increases the noise in the decision signal and increases the leak, while delay-period inactivation disrupts the effect of this decision signal on the behavioral choice.

In general, the experiments are well-designed and the figures presented clearly. The results represent an advance over the authors' previous work, which used pharmacology and calcium imaging in Purkinje cells (ref. 17), but did not reveal the trial- or trial-epoch-specific effects of cerebellar perturbation.

Major points

How frequently did animals fail to lick in either direction? The authors should indicate, for each condition, the fraction of trials with no licks. Is it the case that the "Fraction R choices" plots (e.g. fig. 1d) display data only from trials in which licks occurred?

We have measured the percentage of lick trials (as a fraction of lick plus no-lick trials) and report this in lines 79-81; the percentage of trials with no licks is equal to 100 minus this number. As we note in the text, the no-lick trial rates were not significantly affected by the perturbation. (The percentage of no-lick trials for the sub-cue-period perturbations was 0.4±0.5%, 0.5±0.4%, 1.0±0.8%; mean±sd for first-third, middle-third, last-third, respectively.)

It is indeed the case that all of our analyses used exclusively trials in which a decision lick was made. We have specified this in the Methods section on lines 427-429.

One might expect the perturbations to change the reaction time statistics, but no difference in mean reaction time between control and laser was observed the the pooled data. Did any individual animals exhibit reaction time differences? The standard deviation is rather large; does this reflect differences between animals? Furthermore, the standard deviation appears to be higher for laser (332 ms) than control trials (222 ms). It would be useful to see the mean and standard deviation of the reaction time for control and laser trials for each individual animal.

The light-on and light-off reaction time data are now shown for each individual subject in the new Supplementary Fig. 5. Reaction time distributions are largely overlapping in all animals, and one animal exhibited increased reaction time with light delivery.

How well did the logistic regression model predict behavior? The authors should assess this on a subset of data not used to fit the model.

We have now added a cross-validated evaluation of logistic regression model performance (lines 71-72, 445-447). The regression model predicts animal choice with approximately 75% and 58% accuracy for light-off and light-on trials, respectively.

More details should be provided for the logistic regression analysis, and the model should be written explicitly in the methods. Were three independent variables (number of peri-cue bins) used, and if so, what were the bin edges?

We have now written out the model on lines 440-445 and included these details. Indeed three independent variables were used, comprising three temporally uniform bins spanning the 3.8-second cue period (i.e. bin edges of 0-1.27 s, 1.27-2.53 s, and 2.53-3.8 s).

The caption for fig. 1e states that success rate is proportional to the sum of the logistic regression weights; why is this true?

We demonstrate this point in Figure R1 below. This relationship is related to the fact that the regression model considers choice as a weighted sum of inputs, transformed by a logit. Because the data are in the approximately linear range of the logit function, the statement about proportionality holds.

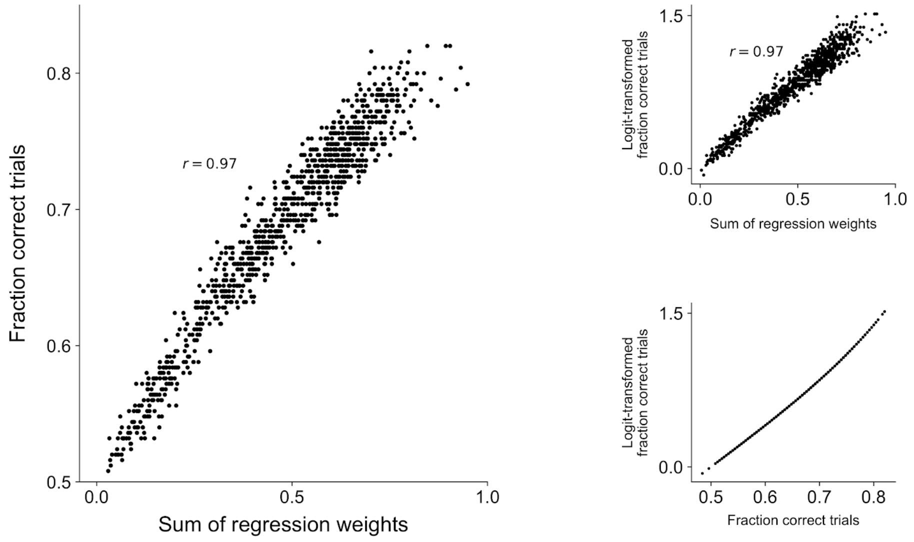

Figure R1: Random batches of trials (n=1000 batches, 250 trials per batch) were drawn from the full behavioral dataset. For each batch, we computed the fraction of correct decisions, and we also fitted the regression model and summed the weights. The left plot shows an approximately linear relationship between the sum of regression weights and fraction correct decisions. Top right: same as left-side panel but with logit-transformed fraction correct decisions (i.e. $\ln\left(\frac{p}{1-p}\right)$) on the y-axis. Bottom right: relationship between fraction correct decisions and the corresponding logit-transformed value for each data point in the other panels.

It would be useful to see the weights for all animals plotted individually for fig. 2 and 1e.

We have now included these plots in the new Supplementary Fig. 7.

Finally, please label the x axis at the three plotted points, rather than 0 and 0.25 s.

The x-axis has now been relabelled at the plotted points in the relevant figures (Fig. 1, 2, S1, S6, S7, S8).

Some aspects of the drift-diffusion model should be explained and justified more thoroughly:

(1) The goodness-of-fit of the drift-diffusion model should be characterized in some way, and should be assessed on a test set not used to fit the model.

We have now characterized the goodness of fit of the drift-diffusion model using a cross-validated procedure (lines 511-514), which is presented in Supplementary Table 1. Specifically, for each of 1000 fit repetitions on subsets (80%) of the data, we used the resulting best-fit parameters to predict animal choice in the remaining 20% of the data not used to fit the model. Based on this procedure, the model predicted animal choice with approximately 73% accuracy. We also include in the same table the Bayesian Information Criterion (lines 514-522) measure for the sake of comparison to other model options.

(2) Is left/right bias the difference between ηR and ηL, or is there an additional bias parameter? If the latter is the case, it should be included in the equation on line 360.

Bias is indeed a separate parameter; whereas η_R and η_L (along with σ_s) parameterize the noise associated with each individual pulse of evidence, bias is time-independent constant offset in the value of *a*.

Because this offset does not contribute to *da/dt* but rather to *a* in a time-invariant manner, we do not include it in the equation mentioned. The meaning of the parameter can be expressed in this form:

$$a(t) = bias + \int_0^t \frac{da}{dt}$$

We include the bias parameter in our fits for the sake of comparisons with other applications of this drift diffusion model, and we have now also included the results of fitting the model without the bias parameter to demonstrate that it does not influence our study conclusions (Supplementary Table 1).

(3) What is the probability of a rightward lick if a(t) > 0 at the end of the trial? It appears to be 1-.5*lapse, but this should be stated.

We now state this on lines 497-498.

(4) Why is a lapse rate parameter needed? It seems that larger values of this parameter reflect a weaker influence of a(t) on behavior. So why not fit the model without a lapse rate and see if the goodness-of-fit is poorer in the perturbation conditions?

We have now also fitted the model without a lapse rate parameter (results in Supplementary Table 1). Indeed the model without the lapse rate parameter more poorly predicts choice in the perturbation conditions than in the light-off condition. Relative to the full model, cross-validated choice prediction is indistinguishable in each condition, while BIC is generally slightly improved as a result of a simpler model with one fewer parameter. We think that while our main conclusions are not strongly altered by this, we agree that it is important to include all of this information and we now do so.

(5) If the model was fit using the BPupsModel package, the github page should be linked.
The fitting procedure should be explained in detail.

The GitHub page has now been linked and the fitting procedure explained on lines 501-510.

Minor points

In fig. 1b (or the supplement), please show a similar panel for laser-on trials.

We now include this panel in the new Supplementary Fig. 4b.

In fig. 1c (or the supplement), the authors should also show laser-on vs laser-off as a scatter plot, in addition to showing the difference.

We now include this panel in the new Supplementary Fig. 4a.

From the discussion: "cerebellar disruption impaired the noise and stability." Would a different word than "stability" be better, as $\lambda < 0$ will increase (not decrease) the stability of a(t) around 0?

We thank the reviewer for noting this confusing terminology and we have changed the phrasing from “impaired the noise and stability” to “impaired the noise and persistent time course” for clarity (lines 145-146).

Reviewer #2 (Remarks to the Author):

This ms follows up on a recent study by the same lab that describes a role for the lateral cerebellum in an evidence accumulation task in mice. Whereas the past study inactivated lateral cerebellum w muscimol, the present study takes advantage of temporal control afforded by optogenetics to attempt to gain insights into the specific role of the cerebellum in this task. The issue of cerebellar roles in non-motor function is timely and of general interest. Because similar decision-making tasks have been well studied in the context of numerous forebrain structures in rodents, the present study could (potentially) elucidate interactions between the cerebellum and forebrain.

1. An important methodological concern is whether the effects of the manipulation are truly related to evidence accumulation or other decision-related processes versus a host of other possibilities more in line with traditional roles of cerebellum in sensori-motor functions. The authors argue that motor behavior is unaffected by the stimulation but they should also check whether the optogenetic stimulation has effects on the movement and position of the whiskers during the stimulation, as might be expected based on previous studies (for example from the Lena group). Video monitoring of whiskers was performed previously for muscimol inactivation but should be reported again here for the optogenetic stimulation.

Another recent study (Brown and Raman) show that air-puffs evoke reflexive whisker protraction in awake mice. Moreover, opto activation of Purkinje cells alters such movements (as well as firing of PCs). If both the stimulus used and the opto manipulation are engaging/altering whisker sensori-motor loops, the interpretation of the results could be very different (i.e. the mice are not just passively integrating sensory evidence).

We agree that the effects of movement and sensory processing are important. In response to the reviewer’s concern, we have now performed video monitoring of whiskers and included it in Supplementary Fig. 10 and Supplementary Movie 2.

Our results are consistent with those of Brown and Raman 2018 (their Figure 4) and of Proville et al. 2014 (their Figure 6), which show a transient effect on some parameters of whisker movement following crus I Purkinje-cell stimulation. Specifically, like their results, we observed brief increases in whisker movement following the onset and offset of light delivery (Supplementary Fig. 10). We hypothesize that these are attributable to attentional shifts associated with light delivery (comparable to that which we observe under normal circumstances at the start and end of the cue period), possibly in combination with transient modulations of the whisker system. We comment on these findings on lines 163-173.

We thank the reviewer for noting this point and we believe the alignment of our new analysis with previous reports is important. These results do not significantly alter our interpretation of the memory-related effects we have characterized. We think the fact that our perturbation does not prevent animals from utilizing evidence presented during or following the light, and that it has effects on stimuli delivered seconds prior to light onset, is best explained using the models we have proposed, rather than as a function of the transient whisker movement modulations observed. In our view, cerebellar involvement in sensorimotor loops is a well-characterized finding that sets the stage for a role in working memory.

2. A recent paper (Gao et al. Nature) report a necessary role for the medial cerebellar nucleus in a whisker-based working memory task. The paper shows that the lateral nucleus is not needed. How does this square with the present results? Presumably, the cortical area activated here projects to the lateral nucleus. A weakness of the present study is that no attempts were made to map the cerebellar regions involved in the task. As far I can tell, the previous paper from the same group did not do this either. Especially in light of the Gao paper, what, if anything, can we conclude regarding how different regions of the cerebellum might contribute to the author's task? The interpretation would be different if activating wide swaths of the cerebellum had exactly the same effect.

We agree that the distinct roles of cerebellar regions is an important question, and systematic optical perturbation studies with laser-scanning systems are likely to shed light on this.

Gao et al. did find preparatory neural activity in the lateral nucleus, but that lesions did not affect choices or movement. We think this is likely explained by important

differences in our tasks: consistent with its proposed roles in higher cognitive function and relationship to prefrontal cortex, the dentate nucleus may be needed for manipulation of working memory contents, such as in the accumulation of evidence in our task. Since the task in Gao et al. does not put strong demands on working memory (substantially shorter memory durations involving a single unchanging stimulus), dentate activity may not have been crucial to execution (while fastigial nucleus may play a coarser motor-preparatory role, consistent with proposed motor roles for more medial cerebellar structures).

We have added discussion of this topic on lines 210-216.

3. I have some questions about the main conclusions. The previous paper ended with: "For example, output signals from the cerebellum may be combined with signals in sensory circuits to control the input gain of sensory information into accumulators elsewhere in the brain. This model would be consistent with observations of cerebellar involvement in gating sensory information (Apps et al., 1997; Ozden et al., 2012) and inputs to working memory (Baier et al., 2014; Sobczak-Edmans et al., 2016). In a second possibility, the cerebellum may modulate dynamics of the accumulation process. Finally, cerebellar signals may modulate activity that converts the accumulator value into a decision. Such a post-categorization influence has been observed in prefrontal regions during evidence accumulation (Erlich et al., 2015). Detailed inactivation studies with high spatial and temporal precision can resolve these alternatives."

(a) I feel that the present ms should give me the answer but I am not sure what it is. This could be a problem with the writing, the experiments, the reviewer or some combination. Perhaps the authors can clarify?

We have added text explicitly linking our previous statements to the results of the current study (lines 181-187). In short, our current results suggest that the cerebellar involvement is not related to input gain (since regression weights are normal during light delivery), but that it does modulate the dynamics of the accumulation process (i.e. the noise and leakiness results). We further elaborate on this below in response to reviewer question 3c.

(b) More specifically, I am not sure why in the model-based analysis of figure 3a the experiments in which light was only given during part of the cue period are not included?

We have now included the best-fit drift diffusion model parameters for the sub-cue-period perturbation trials (Table 1). We do not focus on this analysis because the drift-diffusion model we used is not ideally suited for parameter values that change throughout the cue period of a trial, which is likely to occur in the sub-cue-period perturbation, especially given the effects seen in the regression analyses. Nevertheless, we include these fits to demonstrate for consistency that the primary feature of the middle- and last-third perturbation is picked up as leakiness, i.e. failure to sufficiently weight prior evidence in the trial.

(c) The authors seem to interpret the analysis in fig. 3 to indicate impairment in integration but earlier in the paper they say that surprisingly, integration is fine when the light is on. How are these consistent?

We use “evidence integration” to refer to the entire process of both (a) maintaining evidence in memory and (b) adding new evidence to memory (one cannot integrate without both of these functions intact).

Our statement earlier in the paper, “mice had no difficulty using the evidence presented concurrent with light delivery,” relates only to the latter function, whereas we subsequently show that the former function is impaired.

It is therefore a subset of integration functions which are impaired, and that subset is related to the stability of the existing memory, specified by the parameters σ_a and λ (but not σ_s, which is related to the addition of new information into memory, and that is consistent with the earlier mention of unimpaired usage of evidence presented concurrently with light).

Also, Figure 3 shows a change in the lapse rate in both experimental conditions, however, the authors seem to ignore this, concluding that the deficits in the two conditions are caused by separate processes.

We focused on σ_a and λ because they were significantly affected at the p=0.05 level. We nevertheless now comment on the possible rise in lapse rate in the cue-period-perturbation condition (lines 146-151, 187-190).

4. I found the discussion section of the paper too brief and too vague. Given the large amount of past work in this area, I see this as a major deficiency. Readers will want to understand how the cerebellum fits into existing schemes regarding the neural basis for decision making. Presumably, a major motivation of the study is to understand the interrelations between the cerebellum and forebrain regions, motivating the use of a task that has been heavily studied in the context of the neocortex, as well as the striatum. Vague statements such as "The behavioral effects we characterized here were not observed with perturbations of other brain regions in similar paradigms 3–5,7 ." should be expanded upon such that the reader can understand in reasonable detail how the present results compare to ostensibly very similar manipulations performed in very similar tasks in other brain regions, including PPC, FOF, and dorsal striatum. Even if the answers are not clear, stating clearly what the remaining questions are will be helpful.

We have now added discussion of how our cerebellar findings align with previous reports from decision-making studies in other brain regions (lines 191-201). These include explicit comparisons between our results and those from similar previous studies.

The authors should also comment on how the result of the optogenetic activations used here compare to results they obtained previously using muscimol. This is an important issue given many recent results showing that different manipulations can give unexpectedly different results in the same task.

We now comment on the relationship between our optogenetic and muscimol results (lines 179-187). Compression of the psychometric curve was observed in both perturbations, while most of the other effects characterized in this study were not possible to assess in the pharmacological study due to the lack of temporal resolution and perturbation trial counts.

Reviewers' Comments:

Reviewer #1:
Remarks to the Author:
We commend the authors for an excellent paper. All concerns have been sufficiently handled.

Reviewer #2:
Remarks to the Author:
The authors have addressed my major concerns.

Response to Reviews

We thank the reviewers for their thoughtful and constructive critiques.
Below we address each point, with **reviewer text in bold** and author responses in blue.

Reviewer #1 (Remarks to the Author):
We commend the authors for an excellent paper. All concerns have been sufficiently handled.

Reviewer #2 (Remarks to the Author):
The authors have addressed my major concerns.

We thank the reviewers for their constructive and thoughtful critiques of the work.
